# Phenotypical variation within 22 families with Pompe disease

**DOI:** 10.1186/1750-1172-8-182

**Published:** 2013-11-19

**Authors:** Stephan C A Wens, Carin M van Gelder, Michelle E Kruijshaar, Juna M de Vries, Nadine A M E van der Beek, Arnold J J Reuser, Pieter A van Doorn, Ans T van der Ploeg, Esther Brusse

**Affiliations:** 1 Department of Neurology, Erasmus MC, ‘s-Gravendijkwal 230, 3015 CE Rotterdam, the Netherlands; 2 Centre for Lysosomal and Metabolic Diseases, Erasmus MC, ‘s-Gravendijkwal 230, 3015 CE Rotterdam, the Netherlands; 3 Department of Pediatrics, Division of Metabolic Diseases and Genetics, Erasmus MC-Sophia, Rotterdam, The Netherlands; 4 Department of Clinical Genetics, Erasmus MC, Rotterdam, The Netherlands

**Keywords:** Pompe disease, Glycogen storage disease type II, Lysosomal storage disorder, Acid α-glucosidase, Phenotype, Families, Siblings

## Abstract

**Background:**

Pompe disease has a broad clinical spectrum, in which the phenotype is partially explained by the genotype. The aim of this study was to describe phenotypical variation among siblings with non-classic Pompe disease. We hypothesized that siblings and families with the same genotype share more similar phenotypes than the total population of non-classic Pompe patients, and that this might reveal genotype-phenotype correlations.

**Methods:**

We identified all Dutch families in which two or three siblings were diagnosed with Pompe disease and described genotype, acid α-glucosidase activity, age at symptom onset, presenting symptoms, specific clinical features, mobility and ventilator dependency.

**Results:**

We identified 22 families comprising two or three siblings. All carried the most common mutation c.-32-13 T > G in combination with another pathogenic mutation. The median age at symptom onset was 33 years (range 1–62 years). Within sibships symptom onset was either in childhood or in adulthood. The median variation in symptom onset between siblings was nine years (range 0–31 years). Presenting symptoms were similar across siblings in 14 out of 22 families. Limb girdle weakness was most frequently reported. In some families ptosis or bulbar weakness were present in all siblings. A large variation in disease severity (based on wheelchair/ventilator dependency) was observed in 11 families. This variation did not always result from a difference in duration of the disease since a third of the less affected siblings had a longer course of the disease. Enzyme activity could not explain this variation either. In most families male patients were more severely affected. Finally, symptom onset varied substantially in twelve families despite the same GAA genotype.

**Conclusion:**

In most families with non-classic Pompe disease siblings share a similar phenotype regarding symptom onset, presenting symptoms and specific clinical features. However, in some families the course and severity of disease varied substantially. This phenotypical variation was also observed in families with identical GAA genotypes. The commonalities and differences indicate that besides genotype, other factors such as epigenetic and environmental effects influence the clinical presentation and disease course.

## Background

Pompe disease (OMIM 232300: acid maltase deficiency or glycogen storage disease type II) is an autosomal recessive disorder caused by a deficiency of acid α-glucosidase which leads to accumulation of glycogen in various tissues. The deficiency is caused by mutations in the acid α-glucosidase (GAA) gene (OMIM 606800) [1-7]. The combination of mutations in the GAA gene determines the phenotype to a certain extent [8,9]. A heteroallelic combination of two severe mutations that results in a complete enzyme deficiency causes classic infantile Pompe disease. Soon after birth these infants present with generalized hypotonia, hypertrophic cardiomyopathy, feeding difficulties and respiratory problems. If not treated they usually die before the age of one year [4,10,11]. If one of the two mutations is not fully deleterious and some residual enzyme activity remains, the disease can manifest at anytime during childhood or adulthood. This is referred to as non-classic Pompe disease and patients develop slowly progressive limb girdle weakness and respiratory problems [12,13]. Thus, the GAA genotype is the first level at which clinical heterogeneity arises.

In the Netherlands, about 95% of adult patients with Pompe disease and 68% of affected children under 18 years have the mild and common mutation c.-32-13 T > G in one GAA allele combined with a far more severe mutation in the other allele. c.-32-13 T > G is a so called ‘leaky splice’ mutation, producing only 5-15% of the normal amount of messenger RNA and resulting in a proportional amount of structurally and functionally normal acid α-glucosidase [8,14]. The phenotype of patients with one c.-32-13 T > G mutation combined with a fully deleterious mutation varies widely in age of symptom onset and rate of disease progression despite the similar GAA genotype and haplotype [14,15]. Since siblings with Pompe disease are genetically more identical than non-relatives, we assumed that they would develop more similar phenotypes. Some phenotypical variation within affected families has been described in case reports [16-21].

The aims of our study were to describe the phenotypical variation in a large group of families, to determine to what extent the disease in siblings and families with the same GAA genotype had the same clinical presentation and followed the same course, and to identify possible genotype-phenotype correlations.

## Methods

### Study population

In the Netherlands, all patients diagnosed with Pompe disease are referred to Erasmus MC University Medical Centre, which is the national centre of expertise for this orphan disease. For our study we identified all families with non-classic Pompe disease in which two or three siblings were affected. We used the definition of non-classic Pompe disease as described in a previously published letter [13]. Patients with non-classic Pompe disease have onset of symptoms in childhood or adulthood. Onset of symptoms can also be in the first year of life, however, these patients do not have persisting and progressive cardiac hypertrophy such as in classic infantile Pompe disease. In literature, non-classic Pompe disease is also referred as late-onset Pompe disease, although onset of disease can also be in childhood. All patients were seen between October 2004 and December 2012. The Medical Ethical Committee of Erasmus University Medical Center approved the study protocols.

### Enzyme and mutation analyses

Enzyme activity in fibroblasts was measured with 4-methylumbelliferyl-α-D-glucopyranoside (4-MU) as substrate [14]. GAA sequence analyses were performed using genomic DNA isolated from white blood cells as previously described [22,23].

### Clinical features

At the first visit, a thorough medical history was obtained. Patients were asked at what age they first experienced symptoms and what the nature of these symptoms was. The duration of the disease was calculated from the onset of symptoms to the current age of the patient. Distribution of weakness and specific symptoms such as ptosis, bulbar weakness and scapular winging were recorded. Where applicable, the age at start of wheelchair or ventilator use was recorded.

### Statistical analyses

The data were analysed using SPSS 20. Descriptive statistics were used for all calculations and data are presented as medians with ranges. The following parameters were assessed whether they had an influence on the phenotypical variation in and/or between families: disease duration, gender, genotype, enzyme activity in fibroblasts and co-morbidity.

## Results

### Study population

Among a total of 126 patients with Pompe disease cared for at our centre, we identified 23 families with two or three affected siblings. Because in one family a patient was already wheelchair dependent due to other causes, we did not include this family in our analyses. The common c.-32-13 T > G mutation was encountered in all families; in 12 families in combination with c.525delT (r.0), and in 10 families with another equally detrimental second mutation [24]. Four patients from different families died before they were referred to our centre and these patients were not included because of lack of data. The remaining 22 families comprised 50 patients. The median age at first visit was 49 years (range 0–72 years). None of the patients was being treated with enzyme replacement therapy (ERT) at this time. The characteristics of the study population are presented in Table 1.

**Table 1:** **Patient characteristics**^**a**^

	**Total**
Patients	50
Gender: males (%)	24 (48)
Median age at symptom onset in years (range)^b^	33 (1–62)
Median age at diagnosis in years (range)^c^	39 (0–72)
Current median age in years (range)	53 (5–79)
Mobility (%)	
▪ Ambulant	30 (60)
▪ Walking aids	6 (12)
▪ Wheelchair-dependent	14 (28)
Ventilator-dependent (%)	14 (28)
α-Glucosidase activity in fibroblasts (nmol MU/mg.h)^d^	13 (8–18)
Families	22
Families with genotype (%)	
▪ c.-32-13 T > G/c.525delT (r.0)	12 (55)
▪ c.-32-13 T > G/c.378_379del (p.Cys127LeufsX18)	2 (9)
▪ c.-32-13 T > G/c.925G > A (p.Gly309Arg)	2 (9)
▪ c.-32-13 T > G/other pathogenic mutation	6 (27)

^a^Data are numbers with percentages or medians with ranges. ^b^The median age at onset of symptoms is calculated for 47 patients because three patients are asymptomatic. ^c^One patient was diagnosed at birth because she had a brother with Pompe disease, but she was asymptomatic. ^d^Control range 40–180 nmol MU/mg.h.

### Symptom onset and clinical features

**Figure 1:**
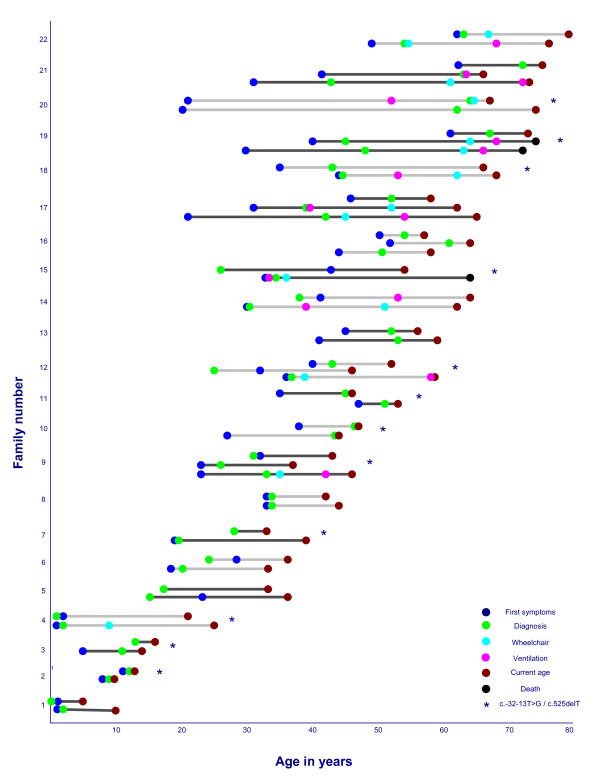
**Course of the disease in 22 families with non-classic Pompe disease.** Families 1–4 are childhood-onset patients and families 5–22 are adult-onset patients. Each dot represents an event during the course of the disease. Families highlighted with * share the same GAA genotype.

Figure 1 gives an overview of the disease course of each family: from symptom onset, to the age of diagnosis and the age siblings became wheelchair or ventilator dependent. The youngest patient developed symptoms in the first year of life, while the oldest patient developed symptoms at the age of 62 years. The median difference in symptom onset was nine years between siblings (range 0–31 years). All siblings within a family developed first symptoms either in childhood (4/22) or in adulthood (18/22). The age of diagnosis varied between 0 and 72 years (median 39 years). The median delay between symptom onset and diagnosis was four years (range 0–43 years) for all patients. The median time between diagnosis of the first sibling and of the second or third family member was one year (range 0–19 years).

Table 2 shows the similarities and differences in clinical characteristics within families. In 13 families all siblings presented with skeletal muscle weakness (i.e. limb girdle weakness or delayed motor development). In one family all three siblings presented with a combination of limb girdle weakness and bulbar weakness. Bulbar weakness comprised dysarthria, dysphagia and chewing difficulties. In the eight remaining families there was a variation in the nature of first symptoms, including three families with a pre-symptomatic sibling (only enzyme and genetic diagnosis, no clinical signs). Specific clinical features seen throughout the course of disease such as ptosis, bulbar weakness and scapular winging were clustered within some families.

**Table 2:** **Clinical characteristics within 22 families**^**a**^

	**Total of families (%)**
Presenting symptoms in all siblings (%)	
Skeletal muscle weakness^b^	13 (59)
Skeletal muscle + bulbar weakness	1 (5)
Variation in first symptoms^c^	8 (36)
Clinical features (%)	
▪ All siblings had a ptosis	4 (18)
▪ All siblings had bulbar weakness	2 (9)
▪ All siblings had scapular winging	1 (5)
Mobility (%)	
All siblings were ambulant	11 (50)
One or two siblings were wheelchair-dependent^d^	10 (45)
All siblings were wheelchair-dependent	1 (5)
Ventilation (%)	
▪ None of the siblings was ventilator-dependent	12 (54)
▪ One or two siblings were ventilator-dependent^d^	9 (41)
▪ All siblings were ventilator-dependent	1 (5)

^a^Data are numbers with percentages. ^b^Limb girdle weakness, delayed motor development. ^c^In three families one sibling was still asymptomatic. ^d^In five families consisting of three siblings, one or two siblings were wheelchair or ventilator-dependent, while the other sibling was not.

### Severity of the disease and possible influencing factors

As shown in Table 2, in approximately 50% of the families there was at least one wheelchair or ventilator-dependent patient. The majority of these wheelchair and/or ventilator-dependent patients (93% and 86%) had a sibling who was ambulant and not using ventilator support at the same age that they became dependent themselves. In one family co-morbidity played a role in clinical variability between siblings, as one sibling acquired kyphoscoliosis after vertebral spondylitis and became wheelchair dependent (Figure 1, family 22). We have added a movie file that illustrates the marked differences in disease course within some sibships [see Additional file 1].

In a subgroup of 11 families representing the largest variation in disease severity, with at least one wheelchair or ventilator-dependent sibling and one ambulant and ventilator-independent sibling, we compared the characteristics of the siblings. The most severely affected sibling had the longest duration of the disease in 64% of these families. Males were more severely affected than females in seven families (64%). In the majority of families (60%), the patient with the highest enzyme activity in fibroblasts was more severely affected.

Twelve families shared genotype c.-32-13 T > G/c.525delT (r.0). The onset of symptoms varied substantially between these families with the same set of GAA mutations (Figure 1); e.g. families 2–4 had symptom onset in childhood, while in families 11, 12, 15, 18 and 19 the first symptoms presented later in adulthood.

## Discussion

This is the first study that describes phenotypical commonalities and variation in a large cohort of families representing 50 patients with non-classic Pompe disease. We confirmed the presence of phenotypical commonalities within sibships, including: symptom onset either in childhood or in adulthood, similar presenting symptoms (in 70% of families), and the occurrence of particular symptoms such as ptosis and bulbar weakness. However, the rate of disease progression varied substantially within some families and also between families with the same GAA genotype.

Infants with classic infantile Pompe disease show a consistent phenotype, also within sibships, while the clinical spectrum of non-classic Pompe disease is much more variable regarding onset and progression of the disease [3,6-8,12]. Since siblings share the same set of GAA mutations and are genetically more related than non-relatives, we expected to find less variation in phenotype within sibships compared to the overall Pompe population. In our 22 Dutch families the presenting symptoms were often the same. As limb girdle weakness is known to be the most frequently occuring initial symptom in Pompe disease, it is not surprising this was found frequently in all siblings of a family [3,5-7,12]. Other symptoms also seemed to cluster within families. Remarkably, bulbar weakness was a presenting symptom in all three siblings of one family. Bulbar weakness has been reported in approximately 25% of adult Pompe patients, but rarely as first symptom (1-2%) [6,12,25]. It was also found in all siblings of a second family but later on in the course of the disease. The same applies to ptosis [26-28]. Ptosis has been reported in 23% of the Dutch adults with Pompe disease [12]. In our cohort of families we found the same percentage. In four families ptosis was present in all siblings. All carried the c-32-13 T > G mutation, but the second GAA mutation differed between the families. The same applied to the two families with bulbar weakness, suggesting a role for (epi)genetic factors in the clustering of symptoms within families.

We also found differences in phenotype within the same family. The median difference in symptom onset between siblings was nine years, with extremes of 20 to 31 years in three families. Since time of symptom onset was based on patients reporting their own history, there may be a recall bias leaving some uncertainty in the differences between time of symptom onset between siblings. However, it is inevitable that large differences will occur within some families. The majority of wheelchair and/or ventilator-dependent patients had an ambulant or non-ventilated sibling, while they had already been using these supportive measures when they were the same age. The duration of the disease (time from symptom onset) could not always explain this difference, since a third of the patients were wheelchair or ventilator-dependent and needed these resources at an earlier stage of their disease than their ambulant or non-ventilated sibling.

We looked for possible factors explaining the observed phenotypical variation between siblings. Siblings who had the disease for longer were often more severely affected. However, this does not explain why these siblings developed symptoms at a younger age and why their disease progressed more rapidly. Gender could play a role since twice as many males as females were more severely affected. Earlier studies on the natural course of Pompe disease also show male gender to be a predictive factor for a more severe respiratory status [12,29]. GAA activity did not explain differences in phenotype, since in only three families did the most severely affected patient have the lowest amount of enzyme activity in fibroblasts. This is in accordance with previously published data on Pompe patients, describing the absence of a correlation between the clinical course and residual activity in patients with the c-32-13 T > G/null genotype [5]. Other co-morbidities contributed to phenotypical differences in only one family.

The substantial differences in age of symptom onset observed in the 12 families with an identical GAA genotype (c.-32-13 T > G/c.525delT (r.0)) suggest that other factors such as variability in genetic background, modifying genes or environmental factors are likely to play a role [5,8,14,16,20,30]. An example of a potential modifying gene is the angiotensin-converting enzyme gene, which has been described as playing a role in modulating phenotype and prognosis in Pompe disease [31,32]. This and other modifying genes might also explain the clear differences between families with the same GAA genotype, and the clustering of symptoms such as ptosis and bulbar weakness in certain families.

## Conclusion

This study in families with non-classic Pompe disease showed that onset of symptoms within a family appeared to be either in childhood or adulthood, presenting symptoms of siblings were often similar and some specific clinical features clustered in certain families. However, the course and severity of disease can vary substantially within some families and between families with the same GAA genotype. This phenotypical consistency and variation within sibships indicates that other factors such as epigenetic and environmental effects influence the course of clinical disease. Additional studies are needed to identify these factors and to determine which prognostic factors will predict the disease course.

## Abbreviations

4-MU: 4-methylumbelliferyl-α-D-glucopyranoside; ERT: Enzyme replacement therapy; GAA: Acid α-glucosidase.

## Competing interests

ATvdP and AJJR have provided consulting services to, and have received research funding from Genzyme Corporation, a Sanofi company, under an agreement between Genzyme and Erasmus MC University Medical Center, Rotterdam, The Netherlands. The other authors declare that they have no competing interests.

## Authors’ contributions

SCAW and CMvG participated in the recruitment of patients, data collection, statistical analyses, data interpretation, and drafting and revising the manuscript for important intellectual content. JMdV and NAMEvdB participated in the recruitment of patients, data collection, and drafting and revising the manuscript for important intellectual content. EB, MEK, AJJR, ATvdP and PAvD participated in the coordination, data interpretation, and revising the manuscript for important intellectual content. All authors read and approved the final manuscript.

## Supplementary Material

Additional file 1 This movie illustrates the marked difference in disease course between two siblings who are both 70 years old. Click here for file
